# Authors’ Reply to: Learning More About the Effects of Gamification on Physical Activity. Comment on “Evaluating the Effectiveness of Gamification on Physical Activity: Systematic Review and Meta-analysis of Randomized Controlled Trials”

**DOI:** 10.2196/38212

**Published:** 2022-05-03

**Authors:** Alexandre Mazeas, Martine Duclos, Bruno Pereira, Aïna Chalabaev

**Affiliations:** 1 Univ Grenoble Alpes SENS 38000 Grenoble France; 2 National Research Institute for Agriculture, Food and Environment (INRAE) Clermont-Ferrand France; 3 Kiplin Nantes France; 4 Department of Sport Medicine and Functional Exploration University Hospital Clermont-Ferrand Hospital G Montpied Clermont-Ferrand France; 5 Department of Biostatistics Unit Clermont-Ferrand University Hospital Clermont-Ferrand France

**Keywords:** behavior change, eHealth, gamification, health behavior, intervention, meta-analysis, mobile phone, physical activity, systematic review, elderly, old adults

We appreciated and read with attention Hung and Kao’s [1] feedback on our recent systematic review and meta-analysis [2], which examined the effectiveness of gamified interventions on physical activity. These authors have pointed out 3 aspects that we will discuss in this letter.

First, they suggest that Paul et al [3] should not have been included in our review as this study is a nonrandomized clinical controlled trial. We agree that this study was nonrandomized. We have in fact mentioned this issue under the Risk of Bias subheading of our Results section: “Overall, 1 study [28] was rated as high risk for sequence generation because assignments were based on recruitment order,” where reference 28 points to Paul et al [3]. This statement was also reported in Multimedia Appendix 2 and was taken into consideration in the summary of findings following the GRADE (grading of recommendations assessment, development, and evaluation) framework, where the quality of evidence for some meta-analyses was downgraded because of the risks of bias in the included studies. Thus, these limitations have been taken into account in our review. Moreover, we would like to emphasize that Paul et al’s [3] study did not have a large heterogeneity contribution and effect size influence as highlighted by our leave-one-out analyses and Baujat plot available in Multimedia Appendix 1. As an example, when omitting this study from the final sample (ie, after sensitivity analyses), we obtained a Hedges *g* of 0.40 (95% CI 0.11-0.75).

Second, Hung and Kao [1] suggest that the total number of hours of gamification performed can have a significant influence and could explain heterogeneity. We cannot agree more on this point since we are convinced that engagement with digital behavior change interventions is necessary to enable an effective intervention. Gamification has often been assimilated into a self-fulfilling process permitting automatic engagement of participants into an eHealth service. However, this is not always the case, which can influence the effect of the intervention. Nevertheless, very few studies measured both engagement and behavioral outcomes in the included studies, preventing us from examining the possible existence of a dose-response. Therefore, we would recommend that future trials should systematically combine measures of engagement in addition to other outcomes. Engagement with the gamified service can be objectively recorded using data from apps and websites (eg, number of logins, time spent per login, number of components accessed), measured via self-report questionnaires (eg, the DBCI Engagement Scale [4]), psychological measures of attention, and qualitative or observational methods.

Finally, Hung and Kao [1] also noted that the results of this meta-analysis may not apply to older adults. If through our meta-regression, the age of participants was not statistically significantly associated with the intervention effect, it is clear that our conclusions cannot be generalized to participants outside the age scope of our review (9-73 years). As they pointed out, few studies have evaluated the effect of gamified interventions on older adults. Future studies should focus on this specific population with specific characteristics.

## Abbreviations

GRADE: grading of recommendations assessment, development, and evaluation
